# Correction to “Mutant p53 proteins counteract autophagic mechanism sensitizing cancer cells to mTOR inhibition”

**DOI:** 10.1002/1878-0261.70017

**Published:** 2025-03-10

Cordani M, Oppici E, Dando I, Butturini E, Dalla Pozza E, Nadal‐Serrano M, Oliver J, Roca P, Mariotto S, Cellini B, Blandino G, Palmieri M, Di Agostino S, Donadelli M. Mutant p53 proteins counteract autophagic mechanism sensitizing cancer cells to mTOR inhibition. *Mol Oncol*. 2016 Aug;10(7):1008–29. doi:10.1016/j.molonc.2016.04.001. PMC5423176.

The article by Cordani et al. contained inadvertent duplications between two western blot images presented in Fig. 4C.

The authors provided original raw data for all experimental replicates, corrected the duplication, and revised the figure panels, which are included here.

All authors agree to this corrigendum and confirm that changes do not affect the conclusions of the article.

The corrected Fig. 4C is reproduced below.

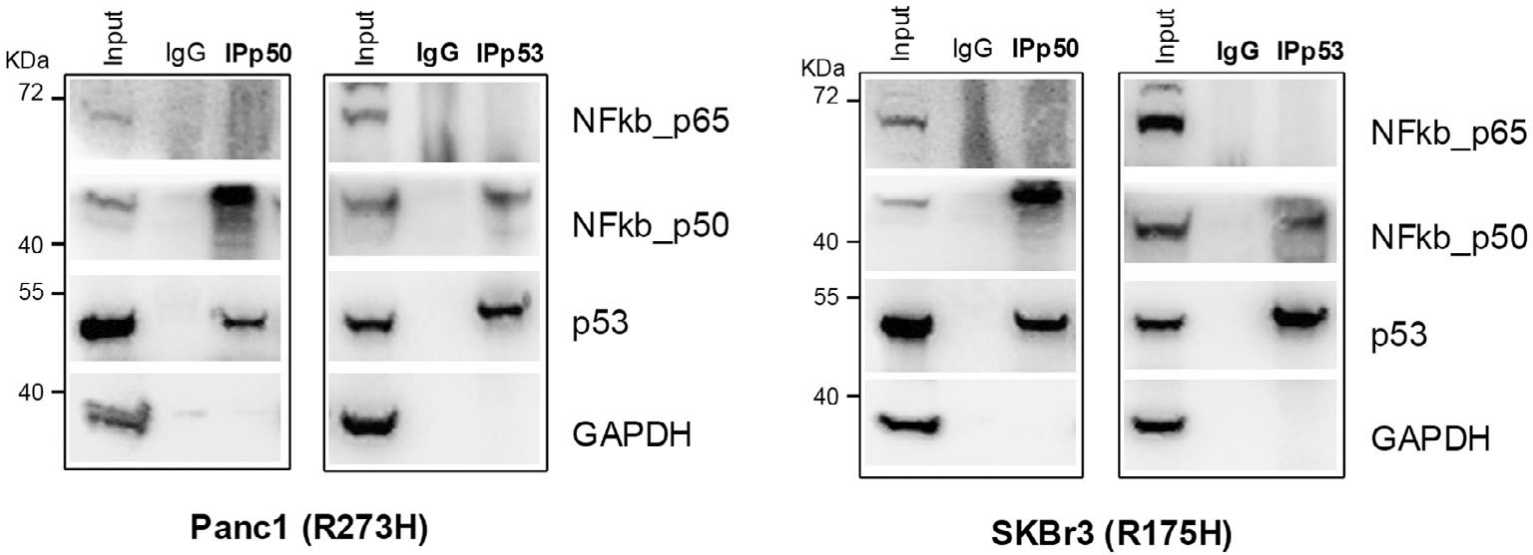

The authors apologize for any inconvenience caused.

